# An extremely prolonged case of drug reaction with eosinophilia and systemic symptoms (DRESS Syndrome) secondary to a penicillin-based antibiotic^☆^

**DOI:** 10.1016/j.abd.2024.07.007

**Published:** 2025-01-13

**Authors:** Florence Robinson, Lucy Webber, Emma Ormerod, Daniel Keith

**Affiliations:** a North Bristol NHS Trust, Bristol, United Kingdom; b Dermatology Department, North Bristol NHS Trust, Bristol, United Kingdom

*Dear Editor,*

Drug reaction with eosinophilia and systemic symptoms (DRESS) is a systemic, delayed cutaneous reaction associated with exposure to a wide variety of medications. The typically associated groups are antiepileptics, antiretrovirals and antibiotics.^1^ DRESS has a large variation in presentation and biochemical characteristics, the syndrome is usually associated with a mobiliform rash, fever, and lymphadenopathy.^2^ The RegiSCAR score has been developed to help quantify the likelihood of a DRESS syndrome diagnosis.^1^ The most common hematological manifestation of DRESS is eosinophilia.^3^ DRESS usually occurs 2‒6 weeks after initiation of the culprit mediation and the symptoms resolve over weeks ‒ months after it is discontinued.^3^ DRESS syndrome is associated with a mortality of 3.8%.^4^

We present the case of a 61-year-old woman with a prolonged presentation of DRESS syndrome. The patient, with a background of marginal zone and follicular lymphoma, was admitted to the hospital with neutropenic sepsis and treated as per protocol with a broad-spectrum antibiotic, tazobactam with piperacillin (tazocin). The patient was discharged home when clinically well but readmitted 2-weeks later with a florid itchy, painful suberythrodermic rash (Fig. 1A‒1B). The associated temperature reached a peak of 40.4 °C. Her eosinophil count on admission was 2.99 and peaked at 7.47 10^9/L. An initial skin biopsy reported a spongiotic psoriasiform inflammatory pattern with impetiginization and eosinophilic infiltrate compatible with DRESS syndrome. No evidence of cutaneous lymphoma was seen. Serum flow cytometry was normal. She was managed initially with prednisolone 30 mg once daily with a slowly reducing course, topical clobetasol ointment and emollients. Despite being off antibiotics and most of her usual medications, she continued to have recall flare-ups of disease with associated smaller peaks in eosinophil count for up to 15-months after the initial onset. A second biopsy during one of these re-flares, about 7-months after the initial onset, confirmed ongoing features consistent with DRESS syndrome with no features of cutaneous lymphoma. Maintenance prednisolone was required at about 20 mg once daily to keep control. Ciclosporin was tried as a steroid-sparing alternative to no effect. Eventually, the clinical and hematological markers of the disease normalized allowing the prednisolone to be tapered and stopped. There has not been a recurrence in 2-years since the last peak in eosinophils.

**Figure 1:**
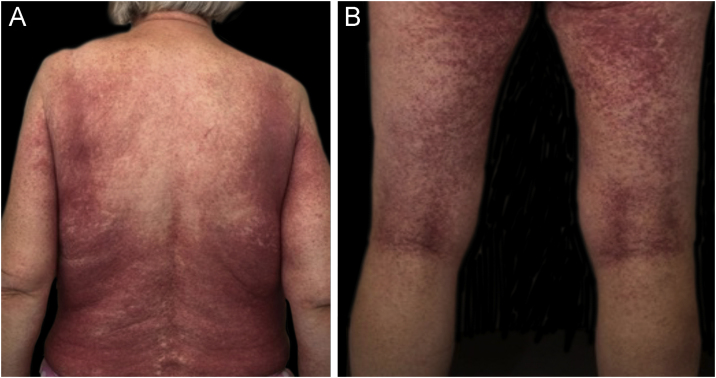
(A) Suberythrodermic rash on back. (B) Suberythrodermic rash on posterior aspect of legs.

After observation of this case, we reviewed the literature for reports of prolonged cases of DRESS syndrome. We identified several papers that reported cases of DRESS syndrome requiring prolonged treatment with steroids.^5^, ^6^ The longest case we reviewed was of a 29-year-old female who developed DRESS syndrome secondary to celecoxib and anti-tuberculosis medication. They reported a relapse in their recovery resulting in a total recovery period of 14-months.^7^ This case was complicated by the development of eosinophilic polymyositis and respiratory failure requiring ventilation. The patient was treated with IV immunoglobulin (IVIG) and steroids. A literature review of DRESS syndrome secondary to antibiotics included 17 cases of DRESS syndrome secondary to co-amoxiclav or tazocin, they reported an 18-day meantime resolution of skin symptoms. The authors acknowledge this to be shorter than the average 3‒6 weeks for resolution of skin symptoms due to tazocin-induced DRESS syndrome.^3^

A prospective case series that specifically reviewed prolonged cases of DRESS syndrome identified out of the 32 evaluated, 7 patients still had persisting features at day 90 and 1 of these 7 still had abnormal liver enzymes indicating ongoing DRESS at 1-year.^8^ This case series reviewed 40 reports and aimed to identify causative factors and features associated with prolonged DRESS syndrome. They found corticosteroid use was the same between those with prolonged DRESS, defined as ongoing DRESS syndrome at day 90, compared to those without.

We believe this is an important case to add to the literature as DRESS syndrome is characterized by an often-prolonged course requiring lengthy treatments with immunosuppressive medication. To the best of our knowledge, this is the longest case of DRESS syndrome recorded in the literature to date.

## Financial support

None declared.

## Authors’ contributions

Florence Robinson: Prepared and wrote the letter for publication.

Lucy Webber: Involved in the treatment of the patient.

Emma Ormerod: Involved in the treatment of the patient.

Daniel Keith: Led the treatment of the patient and reviewed the letter prior to publication.

## Conflicts of interest

None declared.
